# In-Situ Self-Assembling Oligomeric Collagen Scaffold Enhances Vaccine Retention and Vaccine-Induced Humoral Immunity

**DOI:** 10.3390/vaccines13111146

**Published:** 2025-11-08

**Authors:** Juan F. Hernandez-Franco, Sushma Gude, Rachel A. Morrison, Daniela Castillo Perez, Sherry L. Voytik-Harbin, Harm HogenEsch

**Affiliations:** 1 Department of Comparative Pathobiology, College of Veterinary Medicine, Purdue University, West Lafayette, IN 47907, USA; jfhernan@purdue.edu (J.F.H.-F.); casti134@purdue.edu (D.C.P.); 2 Weldon School of Biomedical Engineering, Purdue University, West Lafayette, IN 47907, USA; sgude@purdue.edu (S.G.); morri107@purdue.edu (R.A.M.); 3 Department of Basic Medical Sciences, Purdue University, West Lafayette, IN 47907, USA; 4 Purdue Institute of Inflammation, Immunology and Infectious Diseases, Purdue University, West Lafayette, IN 47907, USA

**Keywords:** vaccination, adjuvant, depot, in situ self-assembly, type I oligomeric collagen, CpG, plasma cells, dendritic cells, antibody response

## Abstract

**Background/Objectives**: Subunit vaccines composed of purified proteins and adjuvants offer excellent safety, but often generate short-lived immunity due to rapid antigen clearance and limited antigen-presenting cell engagement. Sustained, localized delivery of antigen and adjuvant may improve the magnitude and durability of the immune response without compromising safety. This study evaluated an in-situ polymerizing type I oligomeric collagen (Oligomer) scaffold to localize antigen/adjuvant at the injection site and prolong antigen presentation. **Methods**: Mice were immunized intramuscularly with ovalbumin (OVA) and CpG oligonucleotide adjuvant delivered alone or co-formulated with Oligomer. Antibody response and inflammation at the injection site were assessed post-booster at early (Day 32) and late (Day 68) time points. Antigen retention and dendritic cell trafficking to draining lymph nodes were evaluated using fluorescently labeled OVA. **Results**: The Oligomer scaffold retained vaccine antigen at the injection site without eliciting a material-mediated foreign body response. Co-delivery of OVA and CpG within the scaffold enhanced germinal center activity, increased follicular helper T cells and germinal center B cells, and skewed CD4^+^ T cells toward a Th1 phenotype. Humoral responses were greater and more durable, with higher OVA-specific IgG, IgG1, and IgG2a titers and an increased number of bone marrow antibody-secreting cells persisting through Day 68. Antigen-positive dendritic cells, including both resident and migratory subsets, were elevated in draining lymph nodes, indicating enhanced antigen transport. No anti-mouse collagen I antibodies were detected, confirming the maintenance of collagen self-tolerance. **Conclusions**: The Oligomer delivery platform functioned as a localized, immunotolerant vaccine depot, sustaining antigen availability and immune cell engagement. This spatiotemporal control enhanced germinal center responses and generated a more robust, durable humoral immune response, supporting its potential to improve subunit vaccine efficacy while maintaining an excellent safety profile.

## 1. Introduction

Vaccination is a cornerstone of public health, having significantly reduced global morbidity and mortality from infectious diseases. Over the past 50 years, vaccines are estimated to have saved well over 154 million lives worldwide [1]. Advances in molecular biology, biotechnology, and our understanding of disease pathogenesis have enabled the development of molecularly defined subunit vaccines composed of purified proteins or polysaccharides. These well-characterized antigens improve vaccine formulation, quality control, safety, and tolerability. However, they lack intrinsic immunostimulatory signals and are often poorly immunogenic. To overcome this, adjuvants are routinely included to enhance immune responses [2,3,4]. While most licensed vaccines use aluminum-based adjuvants, there is increasing interest in small-molecule adjuvants, such as the Toll-like receptor (TLR) agonist CpG and monophosphoryl lipid A, which more precisely activate innate immune pathways [3,4].

Most vaccines are delivered as intramuscular bolus injections, after which soluble antigens and small-molecule adjuvants rapidly disperse from the injection site. This rapid clearance limits the duration and concentration of antigen and adjuvant exposure, reducing opportunities for sustained engagement with local antigen-presenting cells [5,6,7,8]. Antigen availability is a key determinant of immune activation, as vaccine antigens are captured by dendritic cells (DCs) at the injection site or drained via lymphatics to regional lymph nodes, where DCs present antigen-derived peptides to CD4^+^ T cells. A subset of these differentiates into follicular helper T (Tfh) cells, which are critical for germinal center (GC) reactions and generation of high-affinity antibodies. Concurrently, antigen-specific B cells internalize soluble antigen and either differentiate into short-lived plasma cells or form GCs, where they proliferate, undergo affinity maturation, and ultimately differentiate into long-lived plasma cells or memory B cells under Tfh cell guidance [9,10].

Robust and durable antibody-mediated protection depends on sustained GC activity and the generation of long-lived plasma cells [9,11]. Achieving this immune response typically requires multiple vaccine doses administered over weeks or months. However, since conventional bolus injections provide only brief antigen exposure, lymph node delivery and GC stimulation is limited. Potent adjuvants such as CpG can enhance immune activation, but systemic diffusion may trigger unwanted inflammatory responses, reducing safety and potentially diverting the immune response (“wasted inflammation”) [12]. Recent studies demonstrate that prolonged antigen and adjuvant availability, achieved through continuous delivery (e.g., osmotic pumps or repeated low-dose administration), enhances GC responses and the magnitude and breadth of antibody responses [11,13,14]. These findings underscore the importance of controlling both the spatial and temporal presentation of vaccine components to maximize immune activation while minimizing off-target effects.

Multiple strategies have been pursued to achieve this control [15,16]. Aluminum adjuvants, for example, can adsorb antigens and TLR ligands via electrostatic interactions and ligand exchange mechanisms, creating a depot for slow release [8,12,17,18]. More recently, polymeric nano- and micro-particles have been engineered to encapsulate vaccine components for controlled release and targeted delivery to antigen-presenting cells [19,20]. These approaches have extended into delivery systems such as hydrogels, scaffolds, and microneedle patches, which act as localized immune niches to prolong antigen exposure and enhance immune activation [21,22,23,24]. Despite encouraging preclinical results, translation of such systems has been hindered by formulation complexity, reproducibility, biocompatibility concerns, and manufacturing scale-up. There remains a critical need for vaccine delivery platforms that are simple, user-friendly, biocompatible, and capable of eliciting durable immune responses while minimizing adverse effects.

In this study, we evaluated an in-situ self-assembling (polymerizing) collagen scaffold as a vaccine delivery platform. This two-part system comprised purified type I oligomeric collagen (Oligomer) derived from porcine dermis and a phosphate buffer based self-assembly reagent [25]. Mixing the two solutions initiates polymerization, transforming the solution into a fibrillar collagen scaffold in situ. As a first-in-kind collagen polymer, Oligomer offers several advantages over conventional delivery platforms, including injectability, in-situ scaffold-formation at the injection site, native-like collagen-fibril architecture, tunable scaffold properties, and amenability to computational modeling [26,27,28,29,30]. Its biocompatibility and unique mode of action have been demonstrated across multiple tissue microenvironments, showing no material-induced foreign body response (immunotolerance) and progressive integration and regenerative remodeling into host tissue without inflammatory-mediated degradation [26,29,30,31]. Its safety and biocompatibility are further underscored by FDA 510(k) clearance as a wound management device (product code KGN), establishing a clear pathway for clinical translation.

Using a mouse intramuscular vaccination model, vaccines were formulated with ovalbumin (OVA) as a model antigen, CpG 1826 as a clinically relevant TLR9 agonist adjuvant, and the Oligomer scaffold as a delivery vehicle. Immunogenicity was assessed by quantifying GC-B cells and Tfh cells in draining lymph nodes, CD4^+^ and CD8^+^ T-cell populations in the spleen, OVA-specific antibody isotypes, and OVA-specific antibody-secreting cells in bone marrow at early (Day 32) and late (Day 68) time points. Antigen localization and trafficking were evaluated using Alexa Fluor^TM^ 647-labeled OVA to visualize persistence at injection sites and transport to draining lymph nodes. Histology and immunolabelling were used to characterize the local tissue response. These studies were designed to test the hypothesis that the Oligomer scaffold prolongs local antigen/adjuvant availability, enhances both humoral and cellular immune responses, and provides a non-inflammatory, biocompatible microenvironment that limits systemic inflammatory exposure and preserves the development of protective immunity.

## 2. Materials and Methods

### 2.1. Animal Studies

**Figure 1:**
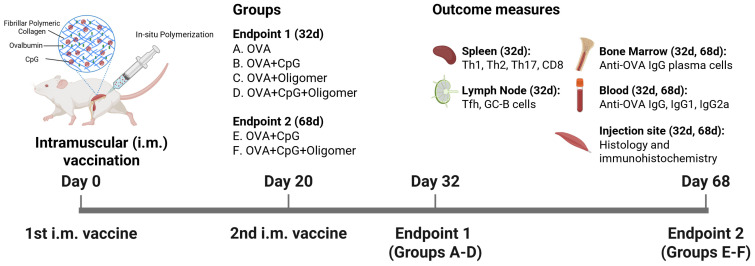
Vaccination schedule and experimental endpoints for assessing the Oligomer vaccine delivery system designed to sustain immunity. Mice were immunized intramuscularly on Days 0 and 20 with OVA ± CpG adjuvant ± Oligomer, and evaluated at early (Day 32) and late (Day 68) endpoints to assess whether the Oligomer scaffold prolongs antigen/adjuvant presentation and enhances sustained immune responses, including T and B cell activation in lymphoid tissues, anti-OVA antibody isotypes in serum, bone marrow plasma cells, and local tissue reactions.

Six- to eight-week-old female outbred Hsd:ICR (CD-1) mice (Inotiv, Indianapolis, IN, USA) were housed in ventilated cages (four animals per cage) with ad libitum access to food and water. Environmental conditions were maintained at 20 ± 2 °C, 50 ± 15% relative humidity, with a 12 h light/dark cycle. Mice were acclimated for one week prior to experimentation. Figure 1 summarizes the study design. Each animal received 100 μL of vaccine (50 μL per two injection sites) via intramuscular injection into the left calf and thigh muscles (Day 0). A booster injection of the same volume was administered into the right calf and thigh muscles on Day 20. Blood and tissue samples were collected on Day 32 or Day 68, corresponding to 12 and 48 days after the second immunization, respectively. An additional cohort of animals was used for antigen localization and trafficking studies, in which vaccines were formulated with Alexa Fluor^TM^ 647-labeled OVA (AF647-OVA; ThermoFisher Scientific, Waltham, MA, USA), and muscle injection sites and draining lymph nodes were collected 48 h after injection. All animal procedures were conducted in accordance with the Guide for the Use and Care of Laboratory Animals and approved by the Purdue University Institutional Animal Care and Use Committee.

### 2.2. Vaccine Preparations

Endotoxin-free OVA and CpG 1826 were purchased from InvivoGen (San Diego, CA, USA) and dissolved in sterile Phosphate-Buffered Saline (PBS). Polymerizable type I oligomeric collagen, purified from porcine skin, was purchased as a sterile, endotoxin-free kit (GeniPhys, Indianapolis, IN, USA) containing an Oligomer solution and a self-assembly reagent. OVA with or without CpG was aseptically mixed with neutralized Oligomer and loaded into sterile insulin syringes immediately prior to injection. The final vaccine doses (100 µL) contained 10 µg OVA, 25 µg CpG and 300 μg Oligomer.

### 2.3. Enzyme-Linked Immunosorbent Assay (ELISA)

OVA-specific IgG, IgG1, and IgG2a titers were measured in serum collected on Days 32 or 68, as previously described [32]. Briefly, 96-well plates were coated overnight at 4 °C with 1 μg/mL OVA. For some experiments, plates were coated with mouse type I collagen (Sigma-Aldrich, St. Louis, MO, USA). Plates were washed with PBST (PBS containing 0.05% Tween-20; Sigma-Aldrich) and blocked for 2 h at room temperature with PBST containing 1% bovine serum albumin (BSA; Sigma-Aldrich). Serially diluted serum samples were added in duplicate and incubated for 1 h at 37 °C. Bound antibodies were detected using peroxidase-conjugated goat anti-mouse IgG (1030-05), IgG1 (1073-05), or IgG2a (1080-05; SouthernBiotech, Birmingham, AL, USA) and developed with 3,3′,5,5′-tetramethylbenzidine (TMB) substrate (Neogen, Lansing, MI, USA). Reactions were stopped with 2 M sulfuric acid, and absorbance was measured at 450 nm (OD450) using the Synergy HT microplate reader (BioTek, Winooski, VT, USA). Final OD450 values were calculated by subtracting the mean OD450 of three blank samples from each test sample. Endpoint titers were defined as the highest dilution at which OD450 ≥ 0.2.

### 2.4. Enzyme-Linked Immunospot (ELISpot) Assay

OVA-specific antibody-secreting cells (ASCs) in bone marrow extracted from tibias and femurs on Days 32 and 68 were quantified by ELISpot assay [32]. Briefly, MultiScreen IP filter plates (MAIPS4510; Sigma-Aldrich) were ethanol-activated, washed, and coated overnight at 4 °C with 10 μg/mL OVA. After washing with PBS, plates were blocked with complete RPMI (RPMI 1640 supplemented with 2 mM L-glutamine, 55 μM beta-mercaptoethanol, 1× non-essential amino acids, 10 mM HEPES, 100 U/mL penicillin, 100 μg/mL streptomycin, and 0.25 μg/mL amphotericin) containing 10% fetal bovine serum (FBS). Serially diluted single-cell suspensions of bone marrow cells were plated in duplicate and incubated for 24 h at 37 °C with 5% CO_2_. Wells were washed and sequentially incubated with biotin-labeled anti-mouse IgG (SouthernBiotech), avidin-HRP conjugate (ThermoFisher Scientific), and 3-amino-9-ethylcarbazole (AEC; Sigma-Aldrich). Spots were quantified using an ELISpot reader (AID Diagnostika, Strassberg, Germany).

### 2.5. Flow Cytometry

Splenocytes were isolated and prepared for intracellular cytokine staining as previously described [32]. Briefly, 1 × 10^6^ cells/well were seeded into 96-well plates in complete RPMI with 10% FBS and 25 μg/mL OVA for 18 h at 37 °C with 5% CO_2_. Following antigen restimulation, cells were centrifuged at 300× *g* for 5 min, resuspended in complete RPMI with 10% FBS, Brefeldin A, phorbol 12-myristate-13-acetate (PMA), ionomycin, and monensin added at concentrations recommended by the manufacturer (BioLegend, SanDiego, CA, USA), and incubated for an additional 6 h at 37 °C with 5% CO_2_. Cells were subsequently washed with 200 μL of Cell Staining Buffer (CSB; BioLegend) and blocked with anti-mouse CD16/32 (clone 93) for 30 min at 4 °C. Cells were labeled using fluorochrome-conjugated anti-mouse CD3ε (clone 145-2C11), CD4 (clone GK1.5), and CD8α (clone 53-6.7) antibodies in CSB for 45 min at 4 °C. After two washes with 200 μL CSB, cells were fixed in 150 μL 4% paraformaldehyde overnight at 4 °C and permeabilized with 200 μL Perm Wash Buffer (BioLegend) for 1 h at 4 °C. Intracellular cytokines were stained in 50 μL permeabilization buffer utilizing fluorochrome-conjugated antibodies against IFN-γ (clone XMG1.2), IL-5 (clone TRFK5), and IL-17A (clone TC11-18H10.1) for 45 min at 4 °C. After two washes with 200 μL CSB, cells were resuspended in CSB for flow cytometric analysis. Cells were isolated from the draining (iliac) lymph nodes and labeled for Tfh and GC-B cells using fluorochrome-conjugated anti-mouse CD16/32 (clone 93), CD3ε (clone 145 2C11), CD4 (clone GK1.5), CD185/CXCR5 (clone L138D7), CD279/PD-1 (clone29 F.1A12), GL-7 (clone GL7), CD45R/B220 (clone RA3-6B2), and CD95/FAS (clone SA367H8) in CSB for 45 min at 4 °C. Cells were washed with CSB and fixed in 4% paraformaldehyde. Migratory and resident DC screening was performed as previously described. Briefly, DCs were isolated from the iliac lymph nodes 48 h post intramuscular immunization with Alexa Fluor^TM^ 647–conjugated OVA (AF647–OVA; ThermoFisher Scientific). DCs were labeled with fluorochrome conjugated anti-mouse CD11b (clone M1/70), CD11c (clone N418), I-A/I-E (clone M5/114.15.2), CD8α (clone 53-6.7), CD103 (clone 2E7), CD207/Langerin (clone eBioRMUL.2; ThermoFisher Scientific), and CD205 (clone 205yekta; ThermoFisher Scientific) in CSB for 45 min at 4 °C. The DCs were washed twice with CSB and fixed in 4% paraformaldehyde. Prior to acquisition, all cells were resuspended in 200 μL of CSB. Flow cytometric acquisition was performed utilizing the Attune NxT flow cytometer (Invitrogen, Waltham, MA, USA), and data were analyzed with FlowJo software (version 10, FlowJo LLC, Ashland, OR, USA). The gating strategies are outlined in Figure S1.

### 2.6. Histology, Immunohistochemistry, and AF647-OVA Imaging

Thigh and calf muscles isolated for light microscopic analysis were fixed in 10% neutral-buffered formalin, paraffin-embedded, and sectioned. Hematoxylin and eosin (H&E) staining was performed for histological evaluation. Adjacent sections underwent antigen retrieval in citrate-based antigen unmasking solution (40 min, steamer), followed by immunolabeling with rabbit anti-mouse CD68 (Cell Signaling, Danvers, MA, USA) or rabbit anti-mouse CD11c (Cell Signaling). Detection used ImmPRESS^®^ HRP polymer kits (Vector Laboratories, Burlingame, CA, USA), with hematoxylin counterstaining. For AF647-OVA localization, muscles were sectioned, placed in OCT embedding medium, and flash frozen with liquid nitrogen for cryosectioning. Histological evaluation was conducted on H&E-stained sections. Cryosections were imaged using a Zeiss 880 upright confocal microscope (Zeiss, Oberkochen, Germany) to visualize AF647-OVA distribution. Images were processed with Imaris (version 8.4.1, Bitplane, Concord, MA, USA).

### 2.7. Statistical Analysis

Statistical analyses were performed in GraphPad Prism v10.4.1 (GraphPad Software, San Diego, CA, USA). Data were analyzed by one-way ANOVA with Tukey’s multiple comparisons test. Antibody titers were log transformed prior to analysis. A *p*-value < 0.05 was considered statistically significant.

## 3. Results

### 3.1. Oligomer Scaffold with CpG Enhances GC Responses Without Altering Baseline Lymph Node Composition

**Figure 2:**
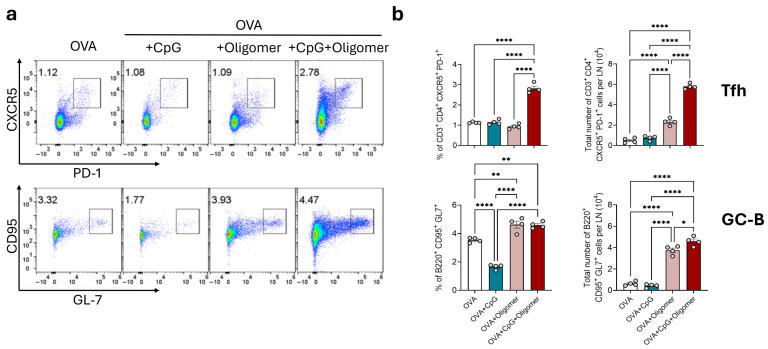
Tfh and GC-B cells in the draining lymph nodes following vaccination with or without the Oligomer scaffold. Draining lymph nodes were collected 12 days after booster immunization and analyzed by flow cytometry. (**a**) Representative bivariate density plots showing Tfh (top) and GC-B (bottom) cells. Numbers indicate the percentage of lymphocytes within the square gating regions. (**b**) Percentage of lymphocytes and number of Tfh and GC-B cells in the draining lymph nodes. Bars represent mean ± SEM (*n* = 4 per group), with individual values shown as circles. * *p* < 0.05; ** *p* < 0.01; **** *p* < 0.0001.

To test the hypothesis that Oligomer-mediated delivery of OVA with CpG enhances early germinal center activity, we quantified Tfh and GC-B cells in draining (iliac) lymph nodes 12 days after the booster immunization (Figure 2). Lymph nodes from mice immunized with OVA + CpG + Oligomer were visibly enlarged, approximately three- to fourfold, compared with those from the other experimental groups. This group also exhibited a significantly higher number of Tfh cells (CD4^+^, CXCR5^+^, PD-1^+^) and GC-B cells (B220^+^, GL7^+^, CD95^+^) compared with the OVA alone group. In contrast, the number of Tfh and GC-B cells in the OVA + CpG, and OVA + Oligomer groups were similar and not significantly different from the OVA group. These findings indicate that the combination of Oligomer scaffold and CpG adjuvant promotes robust germinal center activation in draining lymph nodes.

### 3.2. CpG Drives Th1 Polarization, with Oligomer Scaffold Delivery Maintaining Adjuvant-Induced T-Cell Bias

**Figure 3:**
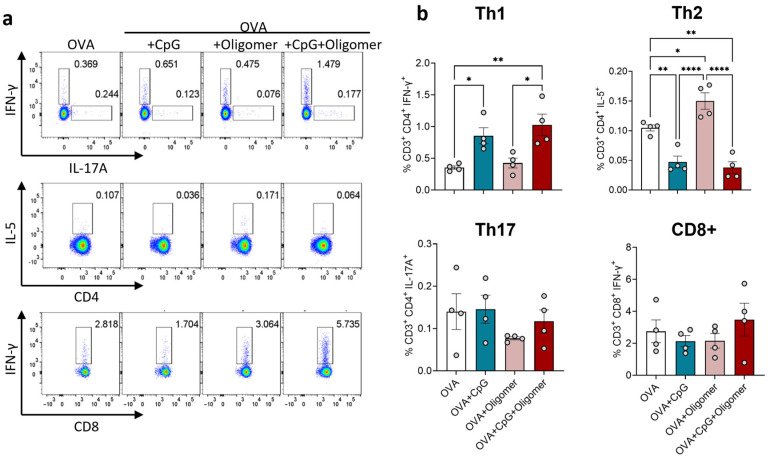
Antigen-specific T cells in the spleen following immunization with OVA ± CpG and/or Oligomer scaffold. Splenocytes were harvested 12 days after the booster dose and analyzed by intracellular cytokine staining after ex vivo re-stimulation with OVA. (**a**) Representative bivariate density plots showing cytokine-producing CD4^+^ and CD8^+^ T cells; numbers indicate the percentage of lymphocytes within the indicated rectangular gating regions. (**b**) Percentages of lymphocytes for Th1, Th2, Th17 T-cell subsets, and activated CD8^+^ T cells. Bars represent mean ± SEM (*n* = 4 per group); individual mice are shown as circles. * *p* < 0.05; ** *p* < 0.01; **** *p* < 0.0001.

To determine the effect of Oligomer scaffold delivery on T-cell polarization, spleen cells were collected 12 days after booster immunization and analyzed by intracellular cytokine staining following OVA re-stimulation (Figure 3). Mice immunized with OVA + CpG or OVA + CpG + Oligomer exhibited a significantly higher percentage of Th1 cells compared with OVA or OVA + Oligomer groups, whereas the frequency of IL-5^+^ Th2 cells was suppressed. The proportion of Th17 cells did not differ between groups, and activated CD8^+^ T cells, as measured by intracellular IFN-γ expression, were not increased in any group. These results indicate that CpG, with or without Oligomer scaffold delivery, skews CD4^+^ T-cell response toward a Th1 phenotype without promoting a detectable increase in cytotoxic T-cell activation. This Th1 bias is consistent with reports that TLR9 agonists, such as CpG oligodeoxynucleotides, promote Th1 polarization and IFN-γ production [33,34].

### 3.3. Oligomer Scaffold with CpG Enhances the Magnitude and Durability of Humoral Immunity Without Inducing Anti-Collagen Antibodies

**Figure 4:**
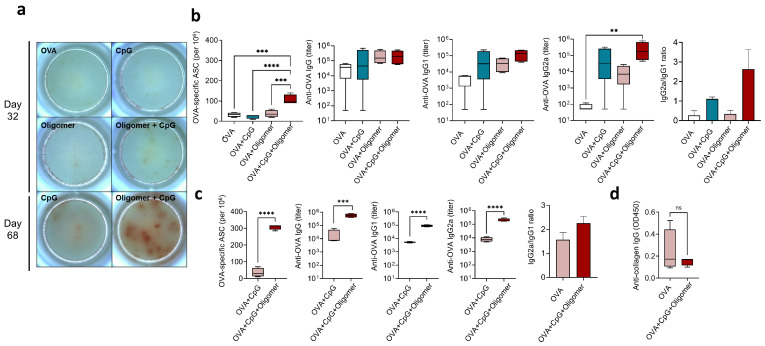
Effect of Oligomer scaffold and CpG adjuvant on the humoral immune response. (**a**) OVA-specific IgG-secreting ASCs in bone marrow quantified by ELISpot, with representative images from one mouse per group. The frequency of ASCs was quantified on (**b**) Day 32 and (**c**) Day 68. Serum OVA-specific IgG, IgG1, and IgG2a levels were measured by ELISA on (**b**) Day 32 and (**c**) Day 68 to evaluate the magnitude and persistence of the humoral response, and the IgG2a/IgG1 ratio was determined. (**d**) Reactivity of serum IgG with type I collagen. Four mice were used per group, and box-and-whisker plots show the median with 25th–75th percentiles. Bars indicate mean ± SEM (*n* = 4 per group); statistical significance was determined by one-way ANOVA with Tukey’s post hoc test. ns, not statistically significant; ** *p* < 0.01, *** *p* < 0.001, **** *p* < 0.0001.

To evaluate the effect of Oligomer scaffold delivery on antigen-specific antibody production, we measured OVA-specific IgG isotypes and quantified ASCs in bone marrow at early (Day 32) and late (Day 68) time points (Figure 4). Twelve days after the booster, total OVA-specific IgG titers were similar among groups. However, mice immunized with OVA + CpG + Oligomer exhibited significantly higher IgG2a levels than all other groups (Figure 4b), consistent with their Th1-skewed T-cell response (Figure 3) as IgG2a is associated with IFN-γ secretion and Th1 differentiation [35,36]. This group also had a greater number of OVA-specific IgG-secreting ASCs in the bone marrow compared with all other groups (Figure 4a,b). To further assess response durability, antibody levels and ASCs were measured 48 days after the booster (Day 68). At this later time point, OVA + CpG + Oligomer immunization maintained significantly higher IgG, IgG1, and IgG2a titers than the OVA + CpG group, along with marked increase in bone marrow ASCs, indicating enhanced persistence of the humoral response (Figure 4c).

Given that the Oligomer scaffold is a polymeric material produced from polymerizable type I oligomeric collagen derived from porcine skin, we next determined whether its use as a vaccine delivery vehicle elicited antibodies against self-collagen. Serum from OVA + CpG + Oligomer-immunized mice revealed no increase in anti-mouse collagen I antibody reactivity compared with OVA-only controls (Figure 4d), indicating that scaffold-vaccine formulation did not disrupt collagen self-tolerance.

### 3.4. Oligomer Scaffold with CpG Enhances DC Uptake and Transport of Vaccine Antigen to Draining Lymph Nodes

**Figure 5:**
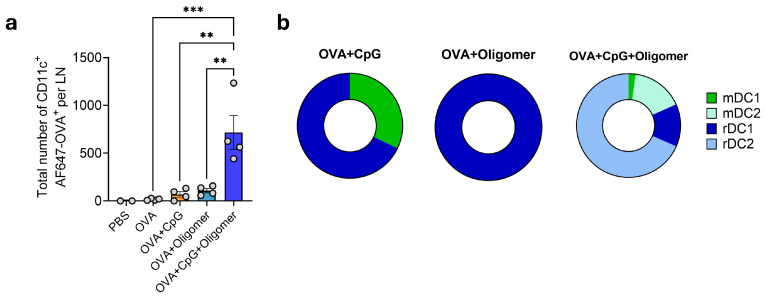
Co-delivery of CpG and OVA within the Oligomer scaffold enhances DC uptake and transport of vaccine antigen to draining lymph nodes. (**a**) Total number of AF647-OVA^+^ CD11c^+^ DCs in the draining lymph node. (**b**) Proportional distribution of OVA-containing migratory mDC1 (CD103^+^ CD11b^−^ CD205^+^ CD207^+^), mDC2 (CD103^−^ CD11b^+^ CD205^+^), lymph node–resident rDC1 (CD8^+^ CD11b^−^ CD205^+^) and rDC2 (CD8^−^ CD11b^+^ CD205^+^) subsets. Data are mean ± SEM (*n* = 4 per group except *n* = 2 for PBS) and are representative of two independent experiments. Statistical significance was determined by one-way ANOVA with Tukey’s post hoc test. ** *p* < 0.01, *** *p* < 0.001.

To assess whether Oligomer scaffold delivery influences antigen capture and trafficking by DCs, mice were immunized intramuscularly with AF647-labeled OVA formulated with CpG, Oligomer, or CpG + Oligomer, and iliac lymph nodes were collected 48 h later. Immunization with OVA + CpG or OVA + Oligomer did not significantly alter the number of OVA^+^ DCs compared with OVA alone (Figure 5a). In contrast, the OVA + CpG + Oligomer group showed a marked increase in total OVA^+^ DCs in draining lymph nodes (Figure 5a). Analysis of DC subsets revealed increases across all populations, with the largest enrichment observed in lymph node-resident DCs (Figure 5b). These findings indicate that CpG and Oligomer scaffold delivery act synergistically to enhance antigen capture and delivery by DCs to lymphoid tissue.

### 3.5. Oligomer Scaffold Enhances Antigen Retention and Supports Vaccine-Induced Immune Cell Recruitment at the Injection Site

**Figure 6:**
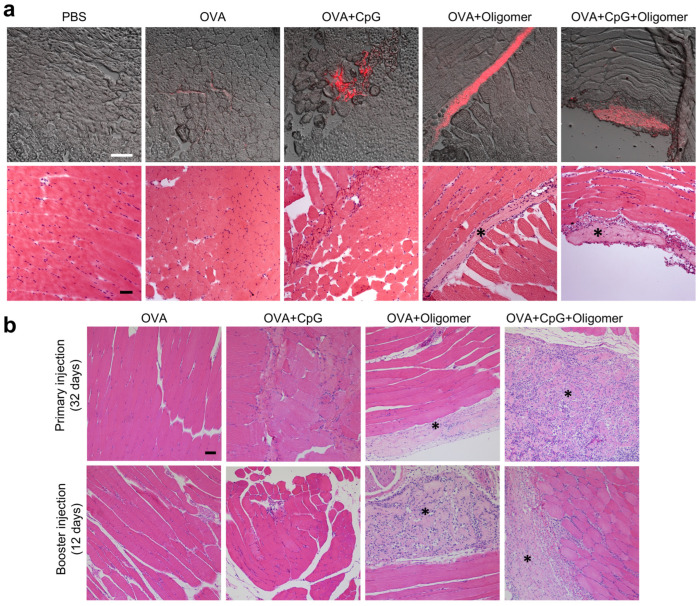
Antigen retention and local tissue responses at injection sites with Oligomer scaffold vaccination. (**a**) Representative confocal images of fluorescently labeled OVA (top; red fluorescence) and corresponding H&E-stained sections (bottom) of hindlimb muscles 2 days after primary injection. Images show group-dependent antigen retention and early cellular infiltration. (**b**) H&E-stained sections at 32 days after the primary injection (top) and 12 days after the booster injection (bottom), highlighting differential cell infiltration patterns. The Oligomer scaffold appears as light eosinophilic material (*). Scale bars: a = 100 µm; b = 50 µm.

Given the importance of immune cell recruitment and antigen trafficking in driving systemic humoral immunity, local responses were evaluated longitudinally through histological analyses to define antigen retention and innate responses at the injection site. To first assess early antigen retention dynamics, muscles were harvested two days following intramuscular injection of AF647-OVA–containing vaccine formulations. Cryosections were analyzed for fluorescent antigen distribution (Figure 6a, upper panel) and adjacent H&E-stained sections were examined for local tissue response (Figure 6a, lower panel). As expected, no fluorescence signal was detected in the PBS control group. OVA alone produced faint, diffusely distributed fluorescence with occasional modest clusters along the injection tract, while the OVA + CpG group showed dispersed clusters of moderate intensity. In contrast, OVA + Oligomer and OVA + CpG + Oligomer both exhibited localized regions of high-intensity fluorescence, consistent with concentrated antigen retention at the injection site.

Histological analysis of H&E sections corroborated these findings (Figure 6a, lower panel). Two days after injection, minimal cell infiltration was observed in the PBS and OVA-only groups, whereas the OVA + CpG group showed modest localized clustering of immune cells, particularly along the injection tract. The highest levels of cellular infiltration were observed in the OVA + Oligomer and OVA + CpG + Oligomer groups, with immune cells primarily concentrated along the scaffold periphery. Additional immune cells were diffusely distributed in the adjacent muscle, consistent with early, limited antigen and adjuvant diffusion from the injection site. This localized immune-cell recruitment was expected given the depot function of the Oligomer scaffold and likely reflects increased antigen/adjuvant-driven activation resulting from greater local concentrations compared with OVA-only or OVA + CpG controls. Throughout the study, animals maintained normal activity and appearance, with no signs of systemic illness (e.g., hunched posture, rough hair coat, or reduced mobility), indicating that the enhanced local inflammatory response was well tolerated.

Tissue responses were further assessed at later time points, specifically 32 days and 68 days after the primary injection and 12 days and 48 days after the booster. It is noteworthy that injection sites were difficult to consistently identify in all animals due to the small injection volume. As shown in Figure 6b, no lesions or significant immune cell accumulation were observed in OVA-only mice at either 32 days after the primary injection and 12 days after the booster injection. At these same time points, OVA + CpG injections produced small foci of muscle remodeling, characterized by increased satellite cells and occasional fibers with centralized nuclei at the primary site, and modest immune cell infiltration at both sites. Immunolabeling confirmed these findings, revealing a few CD68^+^ macrophages and no identifiable DCs in OVA + CpG groups (Figure S2a,b), consistent with rapid diffusion of the vaccine components and the baseline immune cell population in skeletal muscle [37,38].

In contrast, Oligomer-containing formulations revealed light eosinophilic homogeneous material characteristic of the polymeric collagen scaffold between and adjacent to muscle fibers. The scaffold was occasionally detected in adjacent adipose tissue. In the OVA + Oligomer group, a modest number of macrophages, lymphocytes, and eosinophils were present within the scaffold at Day 32 after injection at the primary injection site (Figure 6b, upper panel; Figure S2), with increased mononuclear infiltration, marked by a greater number of CD68^+^ macrophages, at Day 12 after injection at the booster site (Figure 6b, lower panel; Figure S2a). For this group, a moderate number of CD11c^+^ DCs was detected at the primary injection site at Day 32 and a few scattered CD11c^+^ cells at the booster site (Figure S2b). OVA + CpG + Oligomer mice exhibited the most extensive mononuclear infiltration at the primary injection site on Day 32, with immune cells extending between adjacent muscle fibers (Figure 6b, upper panel). Inflammatory cells were predominantly CD68^+^ macrophages with a few multinucleated giant cells (Figure S2a) and a moderate number of CD11c^+^ DCs (Figure S2b). At the booster site, 12 days after administration of OVA + CpG + Oligomer, inflammation was extensive, with CD68^+^ macrophage accumulation observed throughout the Oligomer scaffold and extending into the surrounding muscle (Figure 6b, lower panel; Figure S2a). By Day 68, no lesions were observed in the muscle of mice injected with OVA + CpG. In the OVA + CpG + Oligomer group, remnants of the Oligomer scaffold with fewer associated inflammatory cells were noted in some mice; however, it remains unclear whether this reflected complete collagen remodeling at this time point or difficulties in reliably identifying the injection site. Collectively, these findings indicate that the Oligomer scaffold functions as a local antigen depot, enhancing antigen retention and vaccine-associated inflammatory cell recruitment, with concentrated CpG driving recruitment of macrophages and DCs at the injection site.

## 4. Discussion

Achieving durable, high-quality immune responses with subunit vaccines remains a challenge in vaccinology. Conventional bolus injections typically fail to sustain the antigen exposure needed for robust GC activity and the generation of long-lived, high-affinity antibodies [13,39]. Numerous synthetic and natural biomaterial delivery systems have been developed to address these limitations. Synthetic polymer-based platforms such as poly(lactic-co-glycolic acid) (PLGA), polyanhydride particles, polyethylene glycol hydrogels, and mesoporous silica scaffolds are valued for their tunable physicochemical properties and controlled release kinetics. However, persistent challenges, including antigen instability during fabrication, storage, and release; inflammatory responses driven by degradation by-products; and formulation-dependent variability in immunogenicity and release kinetics, continue to limit their broader translation [40,41]. For example, Wang and colleagues demonstrated that subcutaneous co-delivery of OVA- and CpG-encapsulated PLGA microparticles supported a depot effect for approximately 20 days with serum IgG2b/IgG2c responses peaking at day 56 [42]. Other investigators have shown that PLGA particle size is a critical determinant of depot formation, release kinetics, antigen-presenting cell interactions, as well as material-induced immunogenicity [41,43]. Similarly, in-situ self-assembling scaffolds formed from mesoporous silica rods have been investigated as an alternative strategy, eliminating the need for complex ex vivo formulation [44]. For these systems, the innate immune response to silica drives rapid infiltration of host immune cells, while the porous microstructure dampens the bolus release of vaccine components. When co-formulated with OVA, CpG, and granulocyte-macrophage colony-stimulating factor and delivered as two doses over a 30-day period, these scaffolds generated humoral responses persisting beyond 60 days [44]. Although these synthetic materials are degradable, they do not integrate or remodel into native tissue, often leaving behind fibrotic tissue or capsules [45].

The Oligomer scaffold described in this study addresses many of these limitations by uniquely combining ease of injection, depot stability, immunological neutrality, homeostatic remodeling characterized by natural collagen turnover, and formulation flexibility and tunability, as summarized in Table 1. Upon injection, in-situ self-assembly of soluble type I oligomeric collagen produces a D-banded fibrillar scaffold closely resembling that found in native tissue extracellular matrix [25]. This native-like microstructure entraps antigens and adjuvants within a cell-permissive architecture that supports diffusive transport of vaccine components, vaccine-driven immune cell infiltration (including CD11c+ DCs), and trafficking to draining lymph nodes. These properties were demonstrated by prolonged retention of OVA at the injection site, localized immune cell infiltration within the scaffold, and increased numbers of OVA^+^ DCs across multiple subsets in draining lymph nodes. Collectively, these features promoted durable Th1-skewed humoral immunity, including elevated IgG2a titers and enhanced GC activity that persisted through Day 68, well beyond the responses achieved with soluble OVA + CpG.

Unlike synthetic carriers that degrade into acidic or inflammatory byproducts, or conventional collagen carriers that resorb through inflammatory pathways [46,47,48], the Oligomer scaffold integrates with host tissue and remodels over time through a homeostatic collagen turnover process consistent with regenerative healing [31]. For conventional materials, degradation is tied to controlled release of vaccine payloads, but this process can also lead to variable kinetics and inflammatory microenvironments that may complicate desired vaccine outcomes [39,41]. Additional studies are warranted to better define the kinetics of scaffold remodeling and vaccine bioavailability, and to assess their dependence on material properties such as fibril density and scaffold volume, as well as adjuvant-dependent immune responses.

Importantly, sera from immunized mice did not show increased reactivity to endogenous collagen, indicating that incorporating vaccine antigens and adjuvants into the scaffold does not elicit autoimmunity. This supports the safety of the Oligomer platform for repeat dosing and clinical translation. The Oligomer polymer platform is already FDA-cleared as a wound management device (510(k), product code KGN), underscoring its safety and regulatory readiness. Moreover, its tunable material properties, including microstructure fibrillar density and volume, allow precise modulation of depot effect and antigen/adjuvant bioavailability, while its physiologic self-assembly conditions enable compatibility with a wide range of vaccine payloads, including proteins, nucleic acids, and even living cells (Table 1).

At the cellular level, OVA + CpG delivered in the Oligomer scaffold exhibited significantly increased numbers of GC-B cells, Tfh, and antigen-specific plasma cells compared with soluble formulations. The sustained GC response observed through Day 68 is particularly significant, as GC reactions are critical for affinity maturation and the generation of long-lived plasma and memory B cells [5,40]. Additionally, the presence of antigen-positive DCs in draining lymph nodes, including resident dendritic cell subsets, indicates that the scaffold enhances both local antigen presentation and lymphatic trafficking, further supporting efficient adaptive immune activation. Although vaccine delivery using the Oligomer scaffold elicited significantly higher antibody responses and IFNγ^+^CD4^+^ T cells, a limitation of this study is the lack of direct evidence demonstrating the effectiveness of these responses against viral or tumor challenges.

## 5. Conclusions

This study demonstrates that the injectable in-situ self-assembling Oligomer scaffold, formulated with OVA and CpG as model antigen and adjuvant, elicits robust and durable Th1-skewed immune responses compared to standard soluble formulations. These responses were characterized by sustained GC activity, prolonged IgG2a titers, targeted immune cell recruitment at the injection site, and enhanced antigen presentation in the draining lymph nodes. The established safety profile, remodeling mechanism, and tunability (Table 1) underscore its potential as a versatile and clinically translatable vaccine platform. Future work will focus on optimizing single-dose regimens, conducting detailed antigen/adjuvant dose–response and safety assessments, benchmarking Oligomer performance against aluminum-based adjuvants using clinically relevant antigens, and a viral clearance or survival study to describe enhanced vaccine efficacy. These studies will further define the long-term durability, tolerability, and breadth of immune responses to support translation for both human and veterinary vaccine applications.

## Figures and Tables

**Table 1:** Key structural, functional, and immunological advantages of Oligomer scaffold for vaccine delivery.

Feature	Benefit	Supporting Evidence
Regulatory clearance and safety	Demonstrates biocompatibility, safety, and translation readiness, with FDA clearance as a wound management device (510(k), product code KGN).	510(k) Number: K250329
Injectability	Supports minimally invasive delivery through small-gauge needles, enabled by in-situ polymerization.	[29]; current study
Tissue integration and homeostatic remodeling	Integrates with native tissue and undergoes homeostatic remodeling consistent with natural collagen turnover, without chronic inflammation, and fibrotic encapsulation.	[26,29,30]; current study
Immunologically neutral	Maintains local tissue health without triggering material-induced immune responses, supporting tunability of vaccine formulation for desired outcomes. Preserves self-tolerance to native collagen, with no autoimmunity detected following vaccination.	[29,31]; current study
Tunable properties with amenability to computational modeling	Enables tailoring of material properties, including fibrillar density and scaffold volume, to modulate depot effect and antigen/adjuvant bioavailability.	[28,29,30]
Formulation flexibility	Accommodates a broad range of antigens, adjuvants, and living cells, enabling diverse vaccine applications under physiological conditions.	[27,29,30]; current study

## Data Availability

The data supporting the conclusions of this article will be made available by the authors on reasonable request.

## Supplementary Materials

The following supporting information can be downloaded at https://www.mdpi.com/article/10.3390/vaccines13111146/s1, Figure S1: Gating strategies for flow cytometry; Figure S2: Oligomer depot effects on local immune cell responses at the injection site.
