# Supplementary material for: In-Situ Self-Assembling Oligomeric Collagen Scaffold Enhances Vaccine Retention and Vaccine-Induced Humoral Immunity
Source: Vaccines (Basel). 2025 Nov 8;13(11):1146. doi: 10.3390/vaccines13111146 (PMC12656848; doi:10.3390/vaccines13111146)
Supplement: Supplementary file 1 [file vaccines-13-01146-s001.zip › vaccines-3926134-supplementary.pdf]

Supplementary Material

In-situ Self-Assembling Oligomeric Collagen Scaffold Enhances Vaccine Retention and Vaccine-Induced Humoral Immunity

J. F. Hernandez-Franco, S. Gude, R. A. Morrison, Daniela Castillo Perez, S. L. Voytik-Harbin, H. HogenEsch

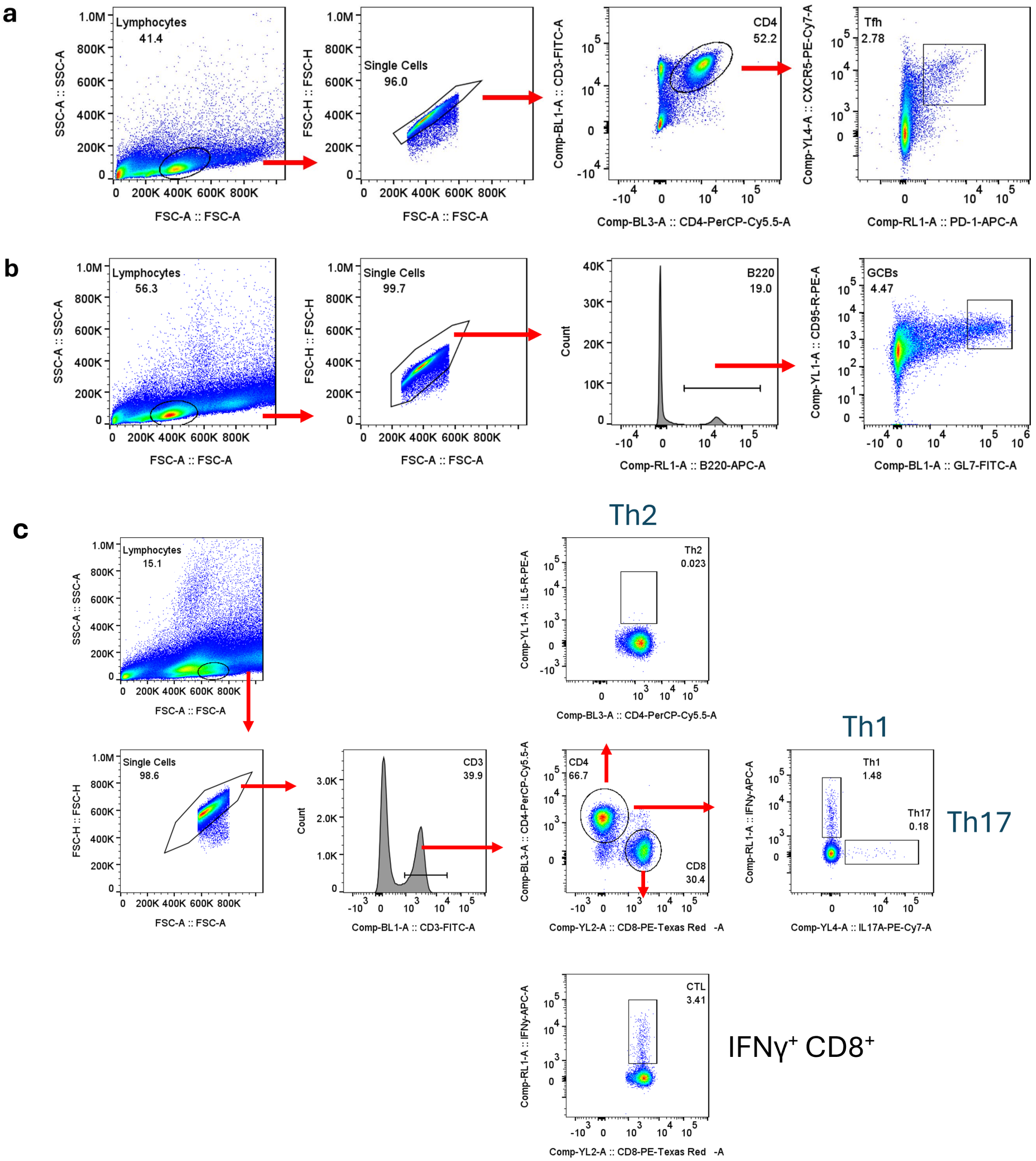

Figure S1. Gating strategies for flow cytometry. (**a**) Tfh cells in the draining lymph node ; (**b**) GC-B cells in the draining lymph node; (**c**) T cell subpopulations in the spleen; (**d**) dendritic cells in the draining lymph node (next page).

d

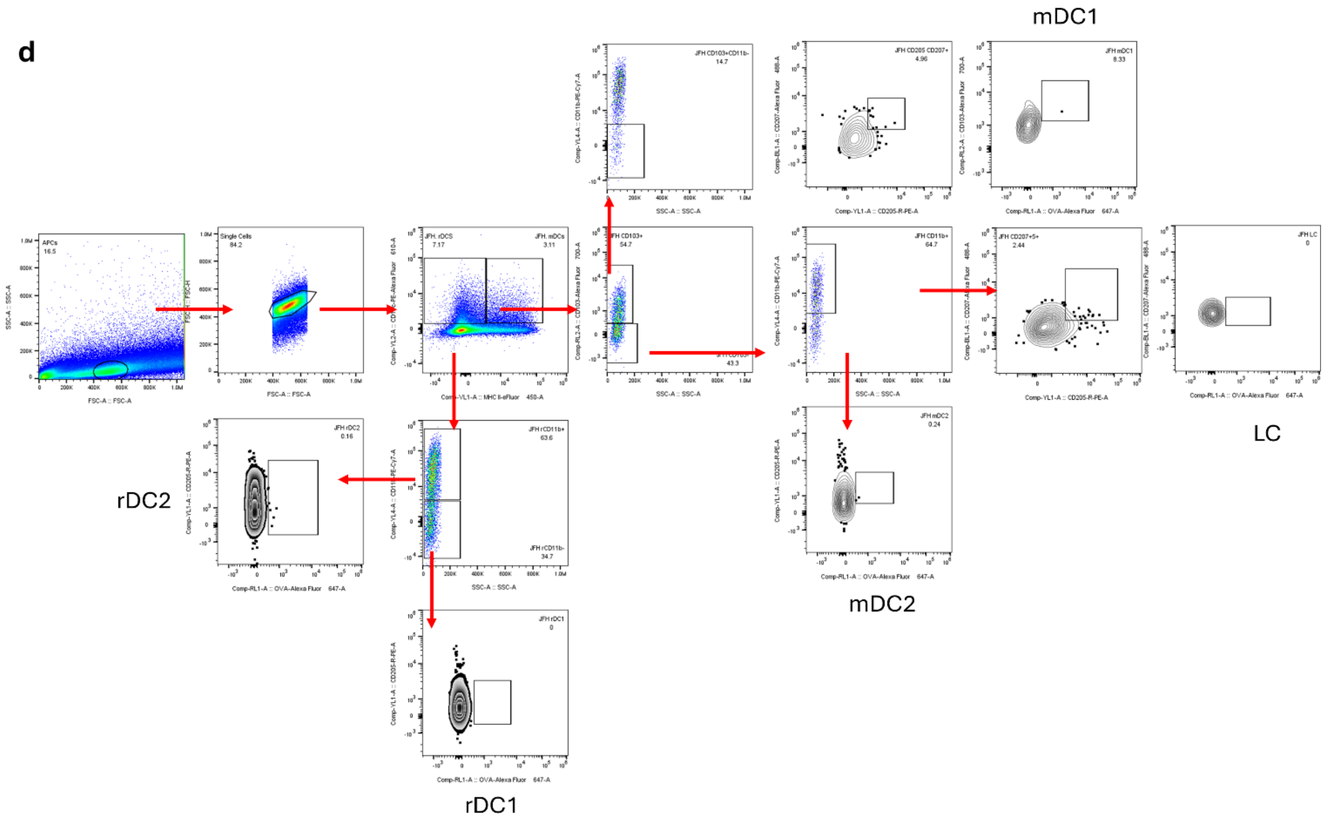

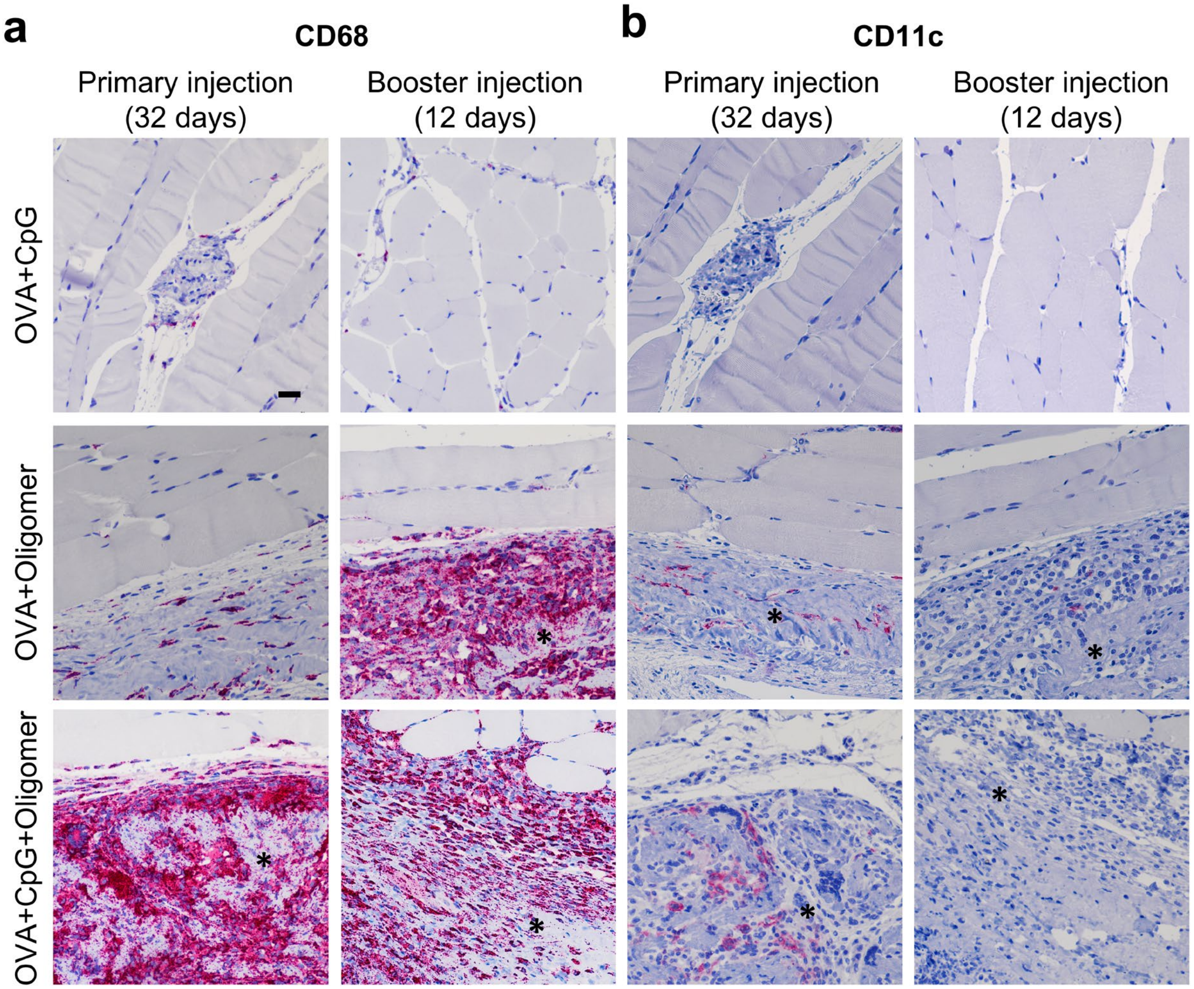

Figure S2. Oligomer depot effects on local immune cell responses at injection sites. Representative CD68 (**a**) and CD11c (**b**) immunolabeled cross-sections of hindlimb muscles showing macrophage and DC recruitment and distribution. Images were collected 32 days after the primary injection and 12 days after the booster injection. Scale bars = 50 μm.
